# Treatment of pathologic fracture following postoperative radiation therapy: clinical study

**DOI:** 10.1186/s40902-015-0032-2

**Published:** 2015-09-29

**Authors:** Chul-Man Kim, Min-Hyeog Park, Seong-Won Yun, Jin-Wook Kim

**Affiliations:** Department of Oral and Maxillofacial Surgery, School of Dentistry, Kyungpook National University, 2175 Dalgubeoldae-ro, Jung-gu, Daegu 41940 South Korea

**Keywords:** Postoperative radiation therapy, Pathologic fracture, Osteoradionecrosis

## Abstract

**Background:**

Pathologic fractures are caused by diseases that lead to weakness of the bone structure. This process sometimes occurs owing to bony change after radiation therapy. Treatment of pathologic fractures may be difficult because of previous radiation therapy.

**Methods:**

In this study, we analyzed clinical and radiographic data and progress of five patients with mandibular pathological fractures who had received postoperative radiation therapy following cancer surgery.

**Result:**

Patients received an average radiation dose of 59.2 (SD, 7.2) Gy. Four of five patients exhibited bone union regardless of whether open reduction and internal fixation (OR/IF) was performed. Patients have the potential to heal after postoperative radiation therapy. Treatment of a pathologic fracture following postoperative radiation therapy, such as traditional treatment for other types of fractures, may be performed using OR/IF or CR. OR/IF may be selected in cases of significant bone deviation, small remaining bone volume, or occlusive change.

**Conclusion:**

Patients have the potential to heal after postoperative radiation therapy.

## Background

A pathologic fracture may occur even under, otherwise, normally tolerated loading forces when a bone has been weakened by an underlying pathologic process. The most common etiology of a pathologic fracture is osteoradionecrosis (49 %), followed by infections (19 %) and malignancy (19 %). The reduction of bony strength may be caused by physiologic atrophy, osteoporosis, or pathologic processes (e.g., cystic lesions, malignant lesions, inflammatory conditions) or be secondary to surgical intervention. Radiation therapy is a useful treatment for head and neck cancer; however, it may also cause pathologic fracture, as irradiation of tissue can promote hypoxia, hypovascularity, and hypocellularity and is thus associated with a number of complications, including xerostomia, loss of taste, limitation of mouth opening, progressive periodontal attachment loss, dental caries, microvascular alterations, soft tissue necrosis, pathologic fracture, and osteoradionecrosis (ORN) [1–5].

Like other fractures, this type of fracture is treatable with open reduction and internal fixation (OR/IF) or closed reduction (CR). However, thus far, there has been no standard treatment protocol for pathologic fracture patients who had undergone bone postoperative radiation therapy because of a malignant tumor; instead, choice of treatment has depended mainly on the experiences of the treating physician. In this study, we sought to establish indications for OR/IF and CR by examining five cases of pathologic fractures after postoperative radiation therapy [6, 7].

## Methods

A retrospective chart review with the medical records, operation notes, and radiographic data was conducted by the authors treated for patients received postoperative radiation therapy in our department from 2003 to 2013. The initial subjects consists of 86 patients (59 males and 27 females) with an average age of 57.8 years (14~82 years) who received postoperative radiation therapy. The causes of radiation therapy were SCC, ACC, osteosarcoma, malignant melanoma, mucoepidermoid carcinoma. We selected pathologic fracture patients who had received postoperative radiation therapy after cancer surgery in our department. We examined factors including the operation process, radiation therapy dose, fracture site, period between the end of radiation therapy and fracture occurrence, treatment method for the fracture (OR/IF or CR), plating method if the patient received OR/IF, complications during healing, and the period from fracture to bone union. Data collected included age, gender, primary disease, site, stage.

## Results

A total of 86 patients received postoperative radiation therapy were collected with an average length of follow up of 4 years. Eight patients were excluded because of data or follow-up loss. Seventy-eight patients remained, 53 patients were male, and 25 were female. The average age was 59 years with a range of 30~79 years. Regarding etiology, 62 (79.5 %), patients were diagnosed with SCC, six (7.6 %) with ACC, three (3.8 %) with osteosarcoma, two (2.6 %) with malignant melanoma, two (2.6 %) with mucoepidermoid carcinoma, one (1.3 %) with fibrosarcoma, one (1.3 %) with verrucous carcinoma, one (1.3 %) with undifferentiated carcinoma.

Sixty-five patients were observed with favorable bone conditions. Some patients had complications. Delayed bone healing, bone exposure, reconstruction plate exposure, and chronic inflammation were observed in two patients. The patients were divided into two groups; the complication rate was based on the radiation dose of 65 Gy. The 65-Gy group showed a higher complication rate than that of the 65-Gy group, but there was no significant difference between the two groups (*p* = 0.75). The details are summarized in Table 1.

**Table 1:** Result of complication status after postoperative radiation therapy according to radiation dose

Radiation dose (total *n*)	Complication	Patients	Total
<65 Gy (51)	Pathologic fracture	3 (5.9 %)	8 (15.7 %)
	Delayed bone healing	2 (3.9 %)	
	Reconstruction plate exposure	2 (3.9 %)	
	Bone exposure	1 (2 %)	
≥65 Gy (27)	Pathologic fracture	2 (7.4 %)	5 (18.5 %)
	Inflammation	2 (7.4 %)	
	Bone exposure	1 (3.7 %)	

Among these five patients (6.4 %), two men and three women reported pathologic fractures. Average patient age was 70.2 (SD, 8.6) years. The most common primary disease was squamous cell carcinoma (SCC, four patients) followed by mucoepidermoid carcinoma (one patient).

**Fig. 1:**
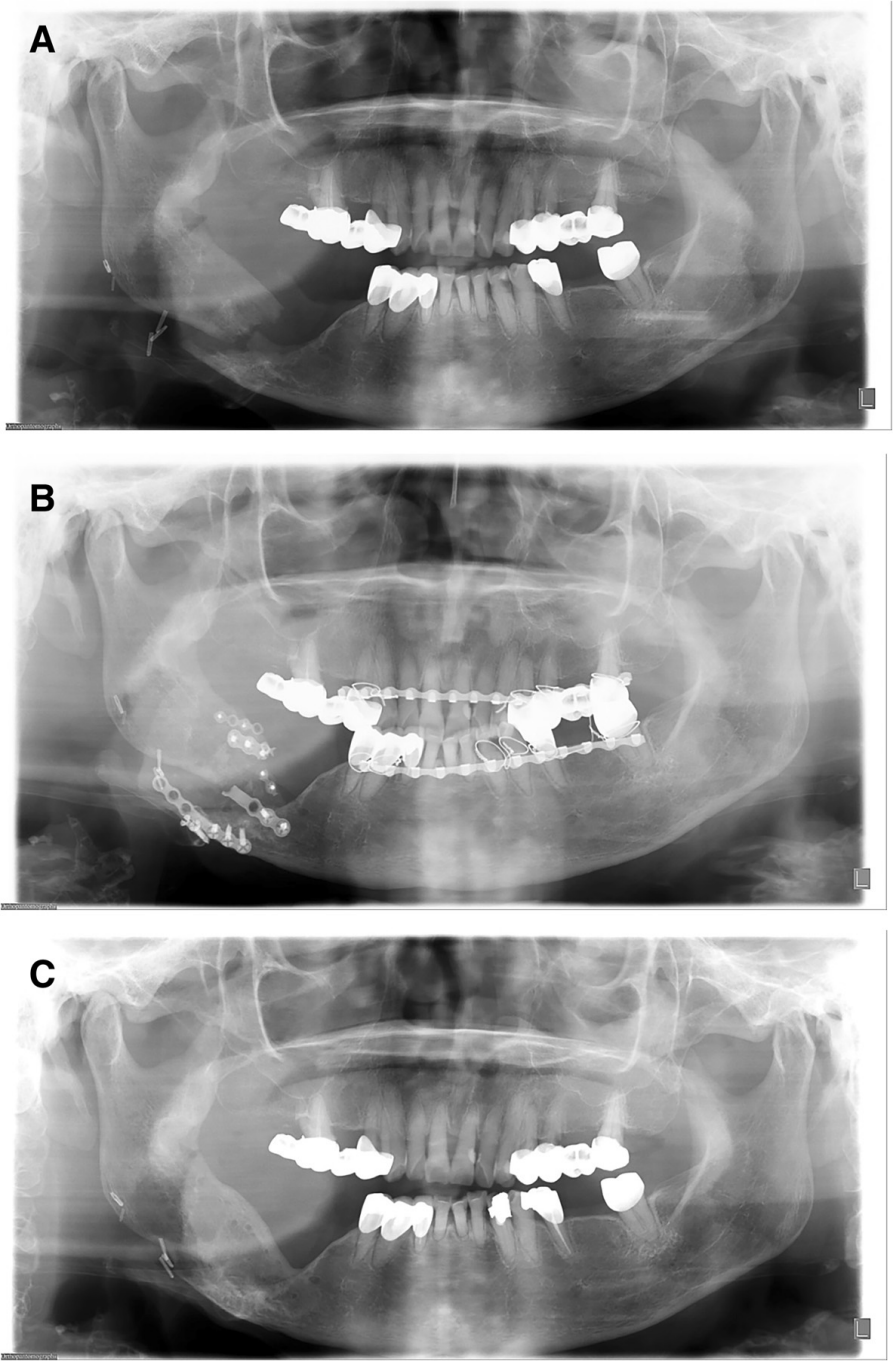
Panorama series of patient 1. **a** Pathologic fracture occurred 3 years after radiation therapy. Iliac bone graft and OR/IF with miniplate was performed on the fracture site. **b** Plate fracture occurred 1 month after OR/IF surgery. **c** Follow up after plate removal. Bone remodeling and recovery of bone continuity were observed

**Fig. 2:**
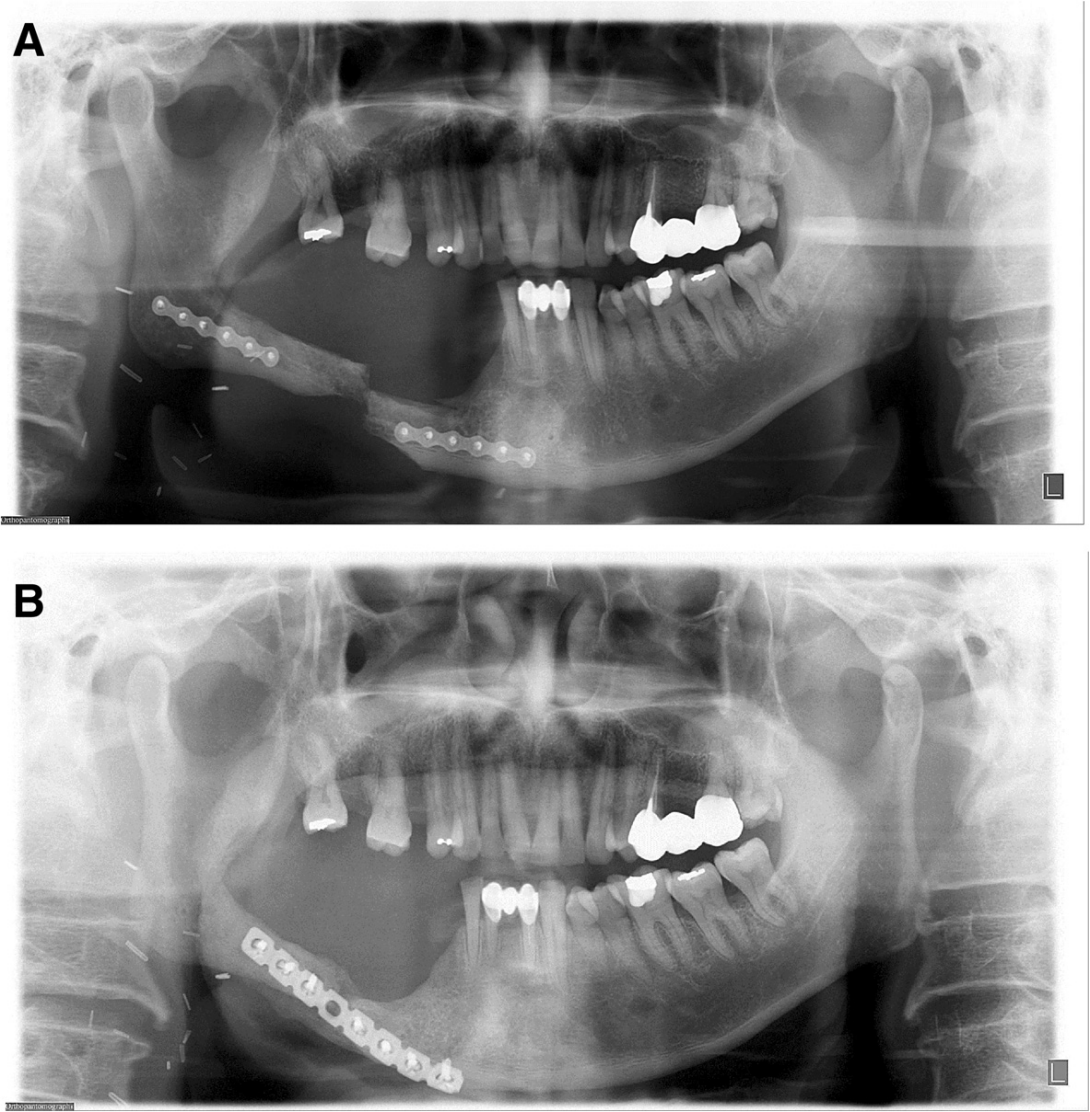
Panorama series of patient 4. **a** Pathologic fracture occurred 9 months after radiation therapy. **b** OR/IF was performed using a reconstruction plate on the fracture site. Nine months after the OR/IF surgery, bone union was observed without plate fracture

The most common sites of a pathologic fracture were the mandibular body and angle, with two patients for each site. There was one patient with a pathologic fracture of the mandibular symphysis. The main complaint was malocclusion or mild pain. Two patients had pus discharge. All patients were dentate state. Reconstruction was performed using miniplate and IBG in patient No. 1 (Fig. 1), reconstruction plate in patient No. 4 (Fig. 2).

The five patients received an average radiation dose of 59.2 (SD, 7.2) Gy. OR/IF was performed on four patients, and conservative care on one patient. In three out of four operation cases, OR/IF was performed using a miniplate; in two of these cases, a non-vascularized iliac bone graft was performed using miniplate fixation. Although plate fracture occurred in two cases, bone union was subsequently observed after control of inflammation. A reconstruction plate was used in one case, which showed successful bone union without plate fracture. More information is given in Table 2.

**Table 2:** Results of ORN due to postoperative radiation therapy reduced pathologic fracture patients

No.	Age/sex	Primary disease	Primary site	Stage			OP on primary lesion	Remaining bone height (mm)	Defect length (mm)	Radiation dose (Gy)	Tx. of fx.	PostOP state	Bone union	Period fx. to healing
				T	N	M								
1	74/F	SCC	Rt Mn post	4	0	0	Marginal resection	18.1	71	60	IBG, miniplate	POD 1-m plate fracture	Union	1Y 7 m
2	80/F	SCC	Lt Mn post	1	0	0	Marginal resection	8.9	41	66	None	–	Union	5Y 4 m
3	76/M	SCC	Lower lip	1	0	0	Surgical excision	4.7	36	54	IBG, miniplate	POD 2-m plate fracture	Union	5 m
4	56/M	SCC	Rt Mn post	1	2	0	Marginal resection	11.1	63	50	Recon plate	Favorable	Union	9 m
5	65/F	MEC	Rt Mn post	2	0	0	Marginal resection	12.3	51	66	Miniplate	Short follow-up (4 m)	Non-union	–

*OP* operation, *Tx* treatment, *Fx* fracture, *F* female, *SCC* squamous cell carcinoma, *Rt* right, *Mn* mandibular, *IBG* iliac bone graft, *POD* postoperative day, *M* male, *Lt* left, *Recon* reconstruction, *MEC* mucoepidermoid carcinoma

## Discussion

Pathologic fracture occurs when a bone defect develops after operation for bone disease. Tyndorf et al. reported that odontogenic cyst and mandibular atrophy were the most common causes of such fractures. Pathologic fracture of the mandible sometimes occurs after surgical removal of these lesions; in particular, the bone defect resulting from marginal resection of malignant tumors or huge benign lesions (e.g., ameloblastoma, keratocystic odontogenic tumors) may be enough to promote development of pathologic fracture [8–10].

Boffano et al. reported that if sufficient bone remained to buttress the fracture, traditional open reduction and internal fixation were performed, in association with cyst enucleation or marsupialization, in almost all reported cases. When remaining healthy bone is insufficient or separated by a large defect, resection of the involved mandibular region, eventually followed by immediate or secondary reconstruction, may be necessary. Abir et al. suggest that in cases where there is no potential for normal union, the bone must be resected until normal, bleeding bone is reached. When sufficient normal bone remains, traditional reduction is performed using rigid fixation. Coletti and Orb also reported that in the few cases in which sufficient bone was left to buttress the fracture, traditional fracture reduction with rigid fixation was employed. As a result, if the potential for bone healing exists, traditional rigid fixation rather than bone resection is recommended [1, 11, 12].

However, pathologic fractures in postoperative radiation therapy patients are different from those in other patients with a pathologic fracture; they often exhibit reduced blood supply and poor condition of the surrounding soft tissue due to irradiation. Moreover, irradiated bone is prone to ORN. In one study, it was reported that 81.8 % of mandibular pathologic fractures were associated with radiolucent lesions. Treatment planning is difficult because of the varying radiation dose of each patient. Many studies have reported that the higher the radiation dose, the greater the extent of tissue damage and the higher the risk of ORN. Several studies have also reported a baseline radiation dose that raises ORN risk. The most reported baseline radiation dose was ≥65 Gy [4, 13].

Moriconi and Popowich suggested that local irradiation of the affected area can also achieve osseous remodeling in some cases. However, treatment decisions in such cases are difficult because of the risk of ORN [14].

In one study, complication occurrence because of delayed bone healing on the fracture line after bone graft in pre-operative radiation therapy patients was reported. Another study reported an increased bone resorption rate (27.9 %) on pre-operative radiation therapy patients after non-vascularized iliac bone graft [15, 16].

In our study, treatment methods were selected according to bone deviation and occlusion stability. In four cases featuring bone deviation and unstable occlusion, we performed OR/IF. Bone healing and remodeling were observed in all OR/IF cases with the exception of one in which bone healing was not observed because of the short follow-up period. The healing periods of the other three cases were 5 months, 7 months, and 1 year and 7 months, with an average healing period of 10 months. These cases had some complications, such as plate fracture; nevertheless, they clearly had healing potential. In two cases of OR/IF, patients had non-vascularized iliac bone graft, and healing was favorable even if they had a plate fracture. Patients who had an operation of OR/IF with iliac bone graft showed more bone formation, about an average of 5.7 mm compared to before fracture. However, the patient group of only OR/IF observed an average of 1.1-mm bone formation than before fracture.

In the CR-treated case, bone continuity was observed after 5 years and 4 months. A relatively high dose of radiation (66 Gy) and unstable occlusion were thought to be the cause of delayed healing.

A total dose of 70 Gy is the standard radiation therapy treatment for head and neck cancer. But, patients in this study were irradiated postoperatively and were assigned to dose levels ranging from 52 to 68 Gy. The average radiation dose was 59.2 (SD, 7.2) Gy; this was lower than the previously mentioned risk baseline of 65 Gy. The lower-than-standard radiation dose in our study may be one reason for the higher rate of bone healing we observed [17, 18].

Among cases of OR/IF in our study, case numbers 1 and 3 experienced plate fracture occurring 1 and 2 months after fixation surgery, respectively. Increased load on the plate due to low remaining bone volume and slow bone healing was likely the cause of fracture; however, bone healing was observed after proper inflammation control and immobilization.

In our study, treatment by OR/IF and CR was found to have similar results. When the treating physician chooses OR/IF, plate selection must be carefully considered; to prevent plate fracture and load sharing, a more rigid plate is recommended rather than a miniplate.

## Conclusion

Patients have the potential to heal after postoperative radiation therapy. Treatment of pathologic fracture following postoperative radiation therapy, such as traditional treatment for other types of fractures, may be performed using OR/IF or CR. OR/IF may be selected in cases of significant bone deviation, small remaining bone volume, or occlusive change. If the operation is chosen, it can be helpful to OR/IF with bone graft for more bone formation. Our study was performed with only five patients. Further study is needed with more patients to get a better precision result.

## Abbreviations

ACC: adenoid cystic carcinoma
C/R: closed reduction
OR/IF: open reduction and internal fixation
ORN: osteoradionecrosis
SCC: squamous cell carcinoma
